# A Neglected Remedy

**Published:** 1888-04

**Authors:** Samuel S. Wallian

**Affiliations:** New York


### A NEGLECTED REMEDY.

BY SAMUEL S. WALLIAN, A. M. M. D., NEW YORK.

The new era which is gradually dawning upon the medical world is based on a more general and systematic study of the use of natural agencies in the removal of disease. The realm of the *vis medicatrix naturæ* is being found broader and more comprehensive than has yet been conceived of. On this mysterious recuperative power, with which every organized being is inherently endowed, and with which all the forces of nature are inherently charged, the intelligent and conscientious physician leans with unhesitant confidence. On his thousand, and ever multiplying, toxic and unnatural drugs he looks with well-grounded and growing distrust. The unreasoning faith in charms, philters and mystery —a heritage from barbaric days—no longer holds him in thrall ; one by one the myths are fading from the medical skies. Every year's experience adds to the growing conviction that the remedial forces of nature are the foundation and crown of all curative processes. All else is open to the suspicion of being patchwork and palliation.

This revival of the use of natural agencies in the prevention and removal of diseased conditions, bids fair to emphasize an epoch in the history of practical medicine. It includes all forms of galvanism and electricity, the various and numberless applications of the forces and influences of heat and cold, light and darkness, motion and rest, labor and recreation, nutrition and waste, æration and ventilation, personal magnetism, passional and psychic relations, moral and social surroundings. It involves a study of climatic peculiarities, of dwellings, dress and occupations, and of every external and internal circumstance and influence which has any natural bearing on the progress or prospects, the success or failure of human existence.

That such a revival—if it be not a veritable revolution—is actively in progress and steadily growing in momentum and importance is everywhere apparent.

Foremost among natural agencies being vigorously discussed, is the general subject of æration and a study of atmospheres ; and this is both proper and timely, since breathing is the prime necessity of all organized or animal life. Hence, the question of æration, in its broadest sense, forms the true basis of all sanitary science. Were it possible to monopolize the supply of atmospheric air, and to dole it out as a marketable commodity, like so much milk or mineral water, how vividly its now but half-appreciated importance would suddenly appear !

On all sides, the habits and customs of civilized life compel us to tolerate, as best we can, a thousand and one drawbacks to strictly hygienic living. Nature has wisely made liberal provision for deviations from an ideal or perfect standard. The climate we inhabit is far from perfect ; alternations of heat and cold, excessive humidity and excessive dryness, all contribute to overtax and depress the vital powers. The food we eat is in no sense ideal ; the water we drink is frequently anything but the pure and sparkling element of which bucolic poets sing ; and the air we breathe is, for the most part, loaded with every conceivable kind of visible and invisible impurities.—germs, spores, organic motes, foul gases, the exhalations of animal bodies and putrifying waste.

Against the vicissitudes of climate we can quite effectually defend ourselves by better clothing and houses, by increased fuel supply, the utilization of steam heat, etc. Of food and drink we have still more direct control, while at best, we partake of these only at stated intervals. Of the air we must constantly partake, whether we will or no. Night and day, early and late, winter and summer, without a single moment's intermission, respiration is the primal necessity.

A little more than a century ago, the air we breathe was considered an elementary and homogeneous body. Among the early Greek philosophers it was held to be the breath of the gods. Jupiter, according to some authorities, was but an emblem of the atmosphere.

Priestley was the sacrilegious surgeon whose scientific scalpel laid bare the anatomy of this fabled deity, who dissected and analyzed the long revered and verse-immortalized divinity. He found him not quite sentient, and not altogether divine; yet a potent and mysterious agency, an æriform spirit, omnipresent and well-nigh omnipotent.

Priestley's discovery of *Oxygen* marked an important epoch in the history of science, and therefore, in the history of the world. In the language of Prof. Draper, a sense of its transcendent importance "will last as long as the world endures."

Liebig declares that since the day of that discovery: "The civilized world has undergone a revolution in manners and customs. The prosperity of empires has increased manifold, and the fortune of every individual has augmented in proportion."

Our own Prof. Youmans adds: "It put an end to old theories, laid the foundation of modern chemical science, and furnished the master key by which man has been enabled to unlock the mysteries of Nature."

Accordingly, the chemical and industrial aspects and bearings of this unparalled revelation in science have already received due recognition, and worked such radical revolutions that chemistry and the arts have accomplished a forward stride, beside which the previous progress of centuries seems insignificant. But we have only recently begun in earnest the careful study of this agent in its bearings upon pathology and the laws of therapeutics. *In this latter field it is destined to work equally as great and radical changes as it has done in the field of industrial and physiological chemistry.*

That this claim is not indulging unreasonable speculation, or anticipating too much, is evidenced by a cursory recapitulation of a few of the commonest facts now authoritatively recognized concerning its nature and possibilities:

*Oxygen* is the most abundant of all the elements, composing one-fifth of the atmosphere, more than one-half of the solid crust of the earth, and eight-ninths of water. It is the most magnetic of all the gases, holding in this respect the same position among gases that is held by iron among metals. (Faraday.)

It supplies an essential and indispensible element toward building and replenishing the tissues, and is itself the active factor in all vital changes.

Functional integrity is maintained by the twin processes, nutrition and depuration. To feed, to renovate ; all the profoundest art of man can do no more. Other effects and results are specious, unnatural and delusive. *These processes are possible only through the direct and constant agency of this subtile element—Oxygen.*

Digestion is a process of vital fermentation. *Oxygen* is the initiator and *sine qua non* of all fermentative processes. It transforms starch into glucose and glucose into alcohol. Assimilation, reparation, elimination, disintegration—all forms of tissue metamorphosis, whether constructive or destructive, progressive or retrogressive, occur and proceed solely by the direct influence, and in the immediate presence of *Oxygen.* Considering the many and indespensible offices constantly presided over by it, the necessity for a normal and adequate supply of this dominant element becomes startlingly apparent.

It instantly changes venous into arterial blood, eliminates waste material from the system, and everywhere and incessantly antagonizes or destroys septic and putrefactive germs. It is the one purifier of the blood, *par excellence ;* at the same time it supplies to the blood and tissues one of the chief elements of nutrition. Exhibited freely, even to excess, it powerfully promotes the dissolution, absorption or elimination of morbid growths and infiltrations ; hence in a therapeutic sense, it is available in nearly every form of chronic disease and in many acute conditions.

The Oxygen of the air is the veritable Midasian wand which transmutes all things it touches into gold, or burns them as dross.

It constantly destroys the myriads of foes to human life which swarm in all climes and in every land, and which keep up their incessant warfare on all that lives.

Even while it glows with life, health and activity, each and every animal organism is slowly but surely evolving its own bane. The toxic *ptomaines* are the result of evolutions and emanations from all vital activities, ard are, at the same time, the very essence and principle of death. Constantly being formed, they are, *by the vital element of the air*, as constantly neutralized, dissolved or destroyed. Whenever the rapidity of their evolution exceeds the power of the air (Oxygen) to eliminate and decompose them, the subject succumbs,—death results. Whatever retards their production or accelerates their elimination, tends to procure and perpetuate health. NON-ELIMINATION *might be truthfully appended to nine-tenths of all the death certificates in the land!*

"The open air is a powerful disinfectant, protecting the gypsy from germs which we vainly fight with all the aid of science." [Dr. B. W. Richardson.]

"There is no true muscle tonic but pure Oxygen." [Prof. W. H. Thompson.]

"The superficial area of the air-cells is eighty-one square meters—*fifty-four times as great as the entire cutaneous surface of the body.* [Prof. Marc See.]

The blood in the pulmonary capillaries travels more rapidly and with less interruption from nervous influences than in the systemic capillaries; *and the whole blood of the body surges through these passages three times every minute.* In the twenty-four hours about *twenty thousand liters of blood are passed through the respiratory organs and brought in immediate contact with the Oxogen of the air.*

Hence it is that epidemics rage in the air; that a large majority of all contagious and infectious diseases are disseminated through the atmosphere and by pulmonary imbibation. We inhale cholera and diphtheria, smallpox and scarlatina, pneumonia, intermitten and, doubtless, typhoid and many other forms of zymotic disease.

Is it, then, at all wonderful or mysterious that through this immense avenue to the human system more can be accomplished, whether for good or evil, than through all other channels combined?

The vapor of alcohol, or the smoke of opium, inhaled, induces more profound and far more rapid intoxication than when these substances are introduced through the ordinary channels. The action of anæsthetics is another familiar proof of the same unappreciated fact.

There are yet difficulties in the way of its general use, but there is no reason why enterprising practitioners, who are not already too severely overworked, or who are not too shiftless or indolent to incur the necessary preparation and painstaking, should longer be without facilities with which to avail themselves of this potent, pleasant and reliable therapeutic agent. Its name has long enough been surrended to advertising quacks. *It is time the profession had rescued it from the smirch of charlatanry which has heretofore attached to it.*

But the past experiments with *Oxygen*, for want of more perfect facilities, have been comparatively crude and unsatisfactory; yet even with the best of these, *patience and painstaking are requisites, for lack of which many experimenters have utterly failed and will continue to fail.*

To do this, better facilities are the first requisite; simpler forms of apparatus for generating the gases; more thoroughness in connection with the carrying out of every minutest detail which will in any way contribute to success.

Too many practitioners have unthinkingly based their estimate of the therapeutic availability of Oxygen on results observed after the desultory use of a few cylinders of ready-made gas, procured from a calcium or oxy-hydrogen light company, or from some mere mechanic and tradesman who does not assume to have any scientific or professional endorsement or responsibility.

Such spasmodic, empirical and haphazard efforts with any remedy invariably fail of other than unsatisfactory or, at best, exceptionally favorable results. Scores of physicians in this city, as elsewhere, are fully convinced that this agent is not only an efficient ally but often a desideratum ; that it is as legitimate and quite as necessary as water, food and sunshine ; yet they have thus far, for the most part, been content to prescribe it, if at all, in the above indicated unsystematic and irrational manner, even entrusting its administration to ignorant or indifferent hands.

In emergencies of a grave character the physician is justified in using the best or only means available ; but in case of chronic ailments which are to be treated for weeks or months, the daily inhalation of carelessly prepared or "commercial gas" will do little or no positive good and may do irreparable harm. To those who are presumed to be familiar with the study of exact methods and the elucidation of minute variations, it ought not to be necessary to emphasize or reiterate this self-evident fact.

